# Integrative investigation of metabolic and transcriptomic data

**DOI:** 10.1186/1471-2105-7-203

**Published:** 2006-04-12

**Authors:** Pınar Pir, Betül Kırdar, Andrew Hayes, Z Ýlsen Önsan, Kutlu Ö Ülgen, Stephen G Oliver

**Affiliations:** 1 Department of Chemical Engineering, Boğaziçi University, Bebek 34342, İstanbul, Turkey; 2 Centre for the Analysis of Biological Complexity, Faculty of Life Sciences, The University of Manchester, Michael Smith Building, Oxford Road, Manchester, M13 9PT, UK

## Abstract

**Background:**

New analysis methods are being developed to integrate data from transcriptome, proteome, interactome, metabolome, and other investigative approaches. At the same time, existing methods are being modified to serve the objectives of systems biology and permit the interpretation of the huge datasets currently being generated by high-throughput methods.

**Results:**

Transcriptomic and metabolic data from chemostat fermentors were collected with the aim of investigating the relationship between these two data sets. The variation in transcriptome data in response to three physiological or genetic perturbations (medium composition, growth rate, and specific gene deletions) was investigated using linear modelling, and open reading-frames (ORFs) whose expression changed significantly in response to these perturbations were identified. Assuming that the metabolic profile is a function of the transcriptome profile, expression levels of the different ORFs were used to model the metabolic variables via Partial Least Squares (Projection to Latent Structures – PLS) using PLS toolbox in Matlab.

**Conclusion:**

The experimental design allowed the analyses to discriminate between the effects which the growth medium, dilution rate, and the deletion of specific genes had on the transcriptome and metabolite profiles. Metabolite data were modelled as a function of the transcriptome to determine their congruence. The genes that are involved in central carbon metabolism of yeast cells were found to be the ORFs with the most significant contribution to the model.

## Background

After the completion of the genomic sequencing of organisms, integrative post-genomic studies and the systems biology approach have emerged with the aim of developing a more complete understanding of cell physiology. Attempts at data integration for the model organism, *Saccharomyces cerevisiae *were reviewed recently [1]. Experimental designs that involve (a) perturbations to elucidate the response of the cell under various conditions, (b) collection of high-throughput data at different functional genomic levels and (c) the use of bioinformatics for integrating data from all three levels of analysis (transcriptome, proteome, and metabolome) constitute the three major steps of a procedure common to all integrative studies.

It is possible to design systems biology experiments in a hypothesis-driven manner, such that the designed perturbations provide the information of interest. Alternatively, question-driven discoveries may be made by observing the effects of an intuitively chosen modification and making use of the extracted information to generate new ideas and hypotheses [2].

Transcriptome data from *S. cerevisiae *growing in chemostats on a glucose medium under carbon, nitrogen, phosphorus or sulphur limitation allowed detection of the genes that were affected by the different nutrient limitations [3]. The genes that were co-regulated under glucose, ethanol, ammonium or phosphate limitation were identified, and genes from the same pathway were shown to be clustered together [4]. Responses to modifications in the growth medium and/or the dilution rate allowed the identification of genes that enable the cells to adapt to various growth conditions [5].

Perturbations can also be introduced by genetic, rather than physiological, means – e.g. by gene deletions. Yeast cells carrying gene deletions have been investigated for various purposes: (a) functional analysis based on discrimination of mutants via metabolic fingerprints [6] or footprints [7], (b) selection of genes encoding organelle-specific proteins [8], (c) building and testing of metabolic pathways [9] and (d) identification of uncharacterized genes and drug targets [10]. These studies have shown that specific changes in the transcriptome or metabolome profiles may occur due to gene deletion. The changes are expected to be more significant when a gene encoding a regulator protein is deleted.

Hap4p was reported to have a function in the regulation of respiration-related genes on the basis of transcriptome data collected during batch growth of yeast cells on glucose, followed by diauxic shift from the fermentation of glucose to the respiratory metabolism of ethanol [11]. The activation mechanism of the Hap2/3/4/5 protein complex has been reviewed by Gancedo, 1998 [12]. The physiology of haploid cells exhibiting *HAP4 *over-expression [13] and the transcriptome profile of haploid *hap4*Δ deletion mutants [14] have also been investigated. *hap4*Δ deletion mutants were reported to be respiratory deficient [8] and deletion of *HAP4 *causes down-regulation of respiration-related genes. In contrast, such genes were expressed at higher levels in *HAP4*-overexpressing strains growing under aerobic conditions. Moreover, an increase in yeast's respiratory capacity was observed due to over-expression of *HAP4 *[13].

In the present study, three types of perturbations that were expected to have an impact on yeast central metabolism, were investigated in chemostat cultures. Changes in growth medium (C- and N-limitations), growth rate (dilution rates) and gene deletions (*hap4 *and *ho*) were the perturbations studied. Transcriptome profiles, biomass, glucose and ethanol concentrations of samples from chemostats operating under steady-state conditions were analysed to show the applicability of the Partial Least Squares (Projection to Latent Structures – PLS) method in the integration of transcriptome and metabolite data.

The PLS method linearly models a set of dependent "response" data with respect to a set of independent "cause" data while repressing both of the sets simultaneously. PLS was recently used to analyse transcriptome data for classification of samples from human tumours [15] and classification of patients for their survival time [16]. In another study, genes expressed periodically within the cell cycle were determined using PLS [17]. Design of experiments and PLS were used for establishing dose- and time-dependent metabolic variations in animals treated with toxic materials [18,19].

## Results and discussion

### Modelling expression levels of ORFs

Linear modelling was used as a filtering tool to eliminate the ORFs with insignificant expression changes in response to the perturbations in growth medium, dilution rate and gene deletion. Mean-centred and scaled (unit variation) expression levels of 6361 ORFs were modelled and p-values were calculated to decide on the significance of the effects of the factors on the expression of the ORFs. For most of the ORFs, the constructed models did not predict a variation more significant than the expected level of random error, thus these ORFs were not included in further analyses. A p-value of 0.05 was used as the threshold, in order to include all ORFs that were affected significantly by the three factors considered in this study.

324 out of 6361 models estimated that at least one of the factors was affecting the expression of the modelled ORF. The growth medium is the factor with most effect on the expression of most of the ORFs (62.1%), followed by dilution rate (26.2%); while gene deletion is the most effective factor for only 11.7% of the ORFs.

### Integration of metabolic and transcriptomic data

**Figure 1:**
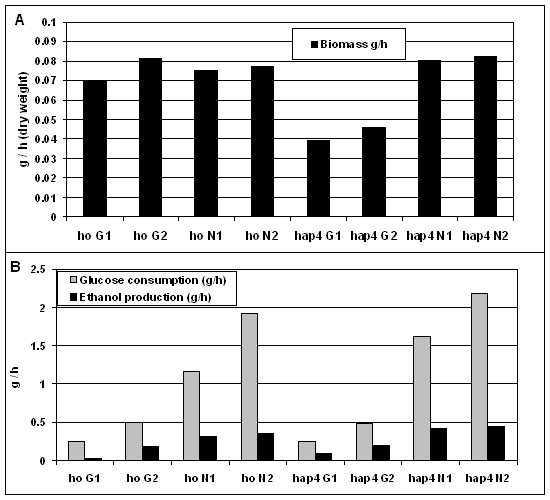
**Biomass and metabolic data**. Biomass concentrations (gram dryweight per hour) were measured at steady state for both the standard (*ho*Δ/*ho*Δ) and mutant (*hap4*Δ/*hap4*Δ) strains grown under glucose limitation at D = 0.1 h^-1 ^(G1) or D = 0.2 h^-1 ^(G2), or under ammonium limitation at the same two D-values (N1, N2). **A**. Glucose consumption rate () and ethanol production rate (■) gram per hour at steady state.

The biomass production rates obtained in chemostats operating at steady state are presented in Figure 1, together with glucose consumption and ethanol production rates. The eight different conditions were selected on the basis of a factorial experimental design (Tables 1 and 2). In the *hap4*Δ/*hap4*Δ deletion mutant, the production rates of both biomass and ethanol are higher under ammonium limitation than under glucose limitation. In contrast, for the standard strain (*ho*Δ/*ho*Δ), only the rate of ethanol production is significantly higher under ammonium limitation as compared to glucose limitation. For both strains, the rate of glucose consumption is elevated under ammonium limitation.

PLS is unable to discriminate between the effects of the different perturbations on the transcriptome; this is because it fails to filter out those transcripts that show significant changes from the vast majority of transcripts that do not change at all (results not shown). Hence, in order to model the metabolic variables as a function of the transcriptome data, and to identify the ORFs that mediate the effects of the perturbations, the partial least squares (PLS) method was applied to the transcriptome of 324 ORFs (X) and metabolic data (Y). Dimensions of the matrices X and Y were 8 × 324 and 8 × 3, respectively, and both matrices were mean-centred and scaled to variance 1 prior to PLS regression. In Table 3, variations represented by the latent variables (LVs) are given in percentages. About 90% of the variance in both data sets is represented in the first three LVs.

**Figure 2:**
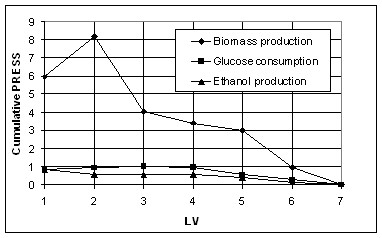
**Cumulative prediction error sum of squares (PRESS) for biomassconcentration, glucose consumption and ethanol production rates**. These values were calculated using a 'leave-one-out' procedure (see text).

Cumulative prediction error sum of squares (PRESS) are plotted to evaluate the prediction power and limitations of the constructed model (Fig. 2). A 'leave-one-out' procedure was used in PRESS calculations; *i.e*., in each step, one of the samples was not included in the model and metabolic profile of the left-out sample was predicted using the model constructed, then the prediction error of that sample was calculated, and the procedure was repeated until all samples had been left out once. The model improves for all three response variables (*i.e*. biomass production, glucose consumption and ethanol production rates) as more latent variables are included (Fig. 2). The only exception was the second LV, since the prediction error for biomass production rate increased when this LV was included in the model. When the third and fourth LVs were included, the cumulative PRESS decreased, indicating that the prediction power of the model was improved – therefore, LV3 and LV4 were included in the following interpretation. The observation of high biomass cumulative PRESS values for all the LVs indicated a low prediction power of the model for the biomass.

**Figure 3:**
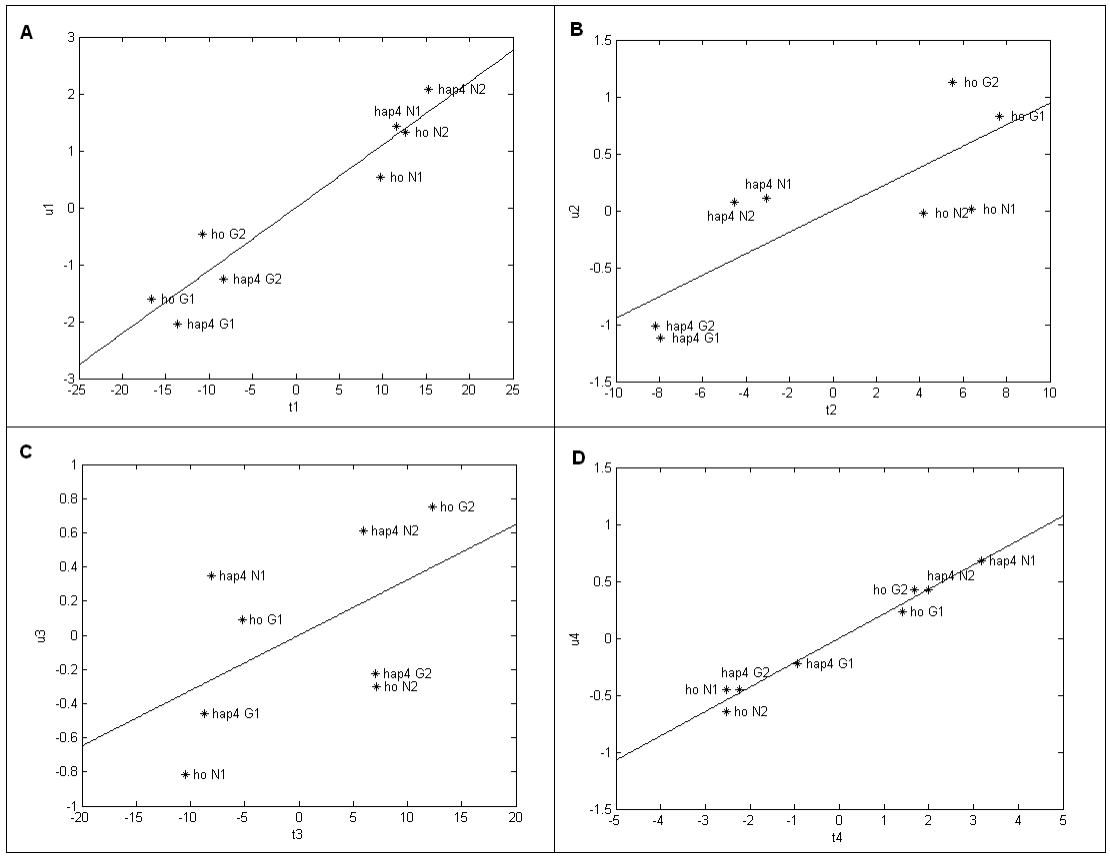
**Comparison of scores for transcriptome and metabolic data**. Scores for the transcriptome (t) and the metabolic data (u) on each LV are plotted against each other. **A**), **B**), **C**), **D**) represent this comparison for the first four latent variables, respectively. In each case, the line shows the modelling capability of the transcriptome on the metabolic data.

In order to see whether the distribution of the samples is linear according to Eq. 5, the scores for the transcriptome (t) and the metabolic data (u) on each LV were plotted against each other (Fig. 3). The distributions of the scores around the fitted lines for LVs 1, 2 and 4 are quite good, thus the modelling capabilities of these LVs are satisfactory (Fig. 3A, B and 3D, respectively), whereas the distribution of scores on LV3 is quite scattered, indicating a weakness of the prediction power of the model for the variation of the metabolic data represented in LV3.

**Figure 4:**
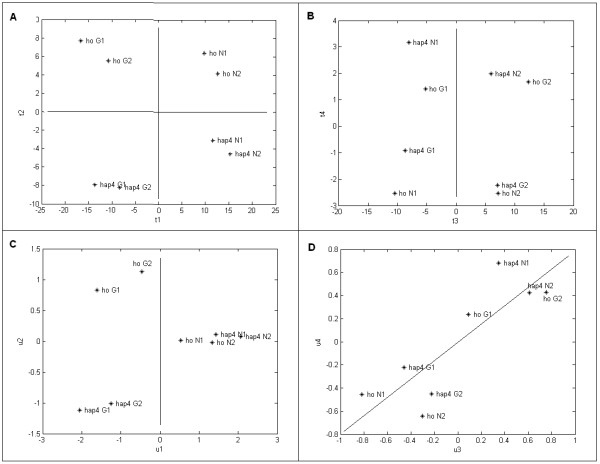
**Scores of transcriptome and metabolic data on first four LVs**. Scores for the transcriptome (t) and metabolic data (u) on each LV are plotted. **A**), **B**) represent projections of the transcriptome samples on the first four latent variables, respectively. **C**), **D**) represent projections of the metabolic samples on the first four latent variables, respectively.

Scores of the transcriptome (t) and metabolic data (u) on the first four LVs are plotted in Figure 4. The first two LVs separate the samples taken from the different media and deletion mutants (Figs. 4A and 4C). The highest variation (56.0% of X and 73.7% of Y), which is represented by LV1, is due to the medium factor and this is followed by the variation in LV2 (13.6% of X and 13.1% of Y) which is due to the gene deletion. In Fig 4A, scores of transcriptome data from ammonium-limited samples are positive on LV1 while scores of transcriptome data from glucose-limited samples are negative. Scores of transcriptome data from *ho*Δ/*ho*Δ samples are positive on LV2, while scores of transcriptome data from *hap4*Δ/*hap4*Δ samples are negative on LV2. Similar discrimination applies for scores of metabolome on LV1 (Fig 4C); however, scores of metabolic variables from *ho*Δ/*ho*Δ and *hap4*Δ/*hap4*Δ mutants can be discriminated on LV2 only if they are from glucose-limited samples. Thus, the effect of gene deletion cannot be modelled by the transcriptome data when the samples are from ammonium-limited fermentors, probably because metabolic variables are not significantly affected by the gene deletions under ammonium-limited conditions, although the transcriptome is significantly affected.

The variation generated by the change in dilution rate was represented by LV3 in transcriptome data (25%, Fig. 4B). Variation generated by the change in dilution rate was represented weakly by LV3 and LV4 in metabolic variables (2.8 and 8.2%, Fig. 4D). Thus, the effect of dilution rate on metabolic variables is not successfully modelled by the transcriptome data.

Each of the latent variables that model the response of the metabolic variables to perturbations using the transcriptome data represents the variation in the data set in one of the perturbations applied in the present analysis (except for the variance generated by dilution rate perturbation, which is represented by two latent variables for the metabolic data). For instance, the projection of samples onto LV1 represents the change that was generated in the sample by ammonium limitation when compared to glucose limitation. The direction of each new variable (LV) in the space is a linear combination of the original variables, i.e. ORFs and metabolites. The direction of an LV is dominated by the variables that respond more than the others and the direction of their response. Thus, an LV can be interpreted as a new composite variable that is the only affected feature in the cell when a certain perturbation is applied. As an exception, for the dilution rate change, two latent variables are needed in order to discriminate the metabolic samples from two different dilution rates.

**Figure 5:**
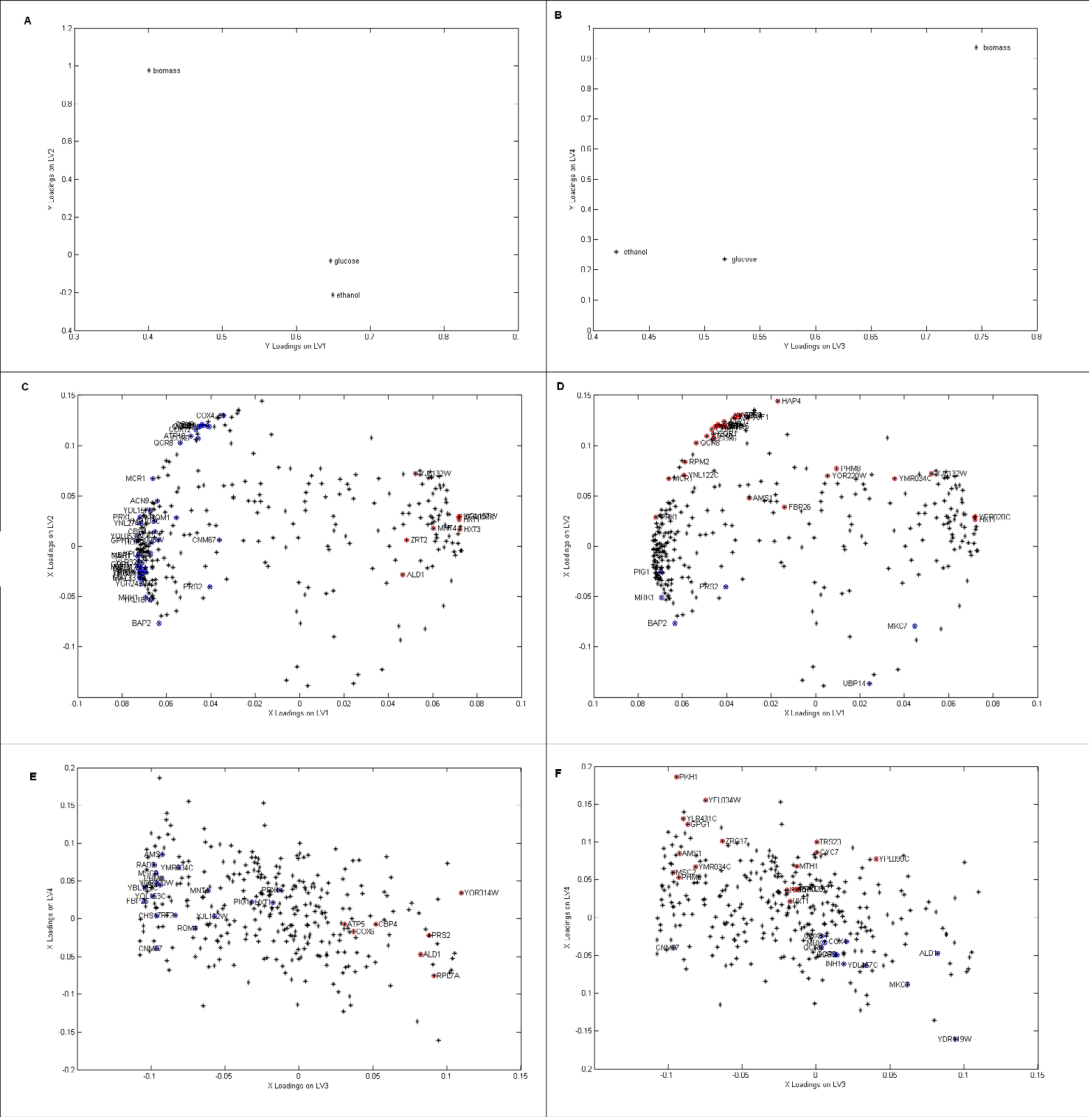
**Loadings of response variables and the ORFs on first four LVs**. oadings of the metabolome (q) on each LV are plotted. **A**), **B**) represent contributions of the samples on the first four latent variables, respectively. **C**), **D**) Loadings of the transcriptome (p) are plotted to visualize the contributions of the ORFs on the first two LVs, ORFs with significant loadings (on LV1 and LV2, respectively) are indicated by red (positive loadings) and blue (negative loadings) circles. **E**), **F**) Loadings of the transcriptome (p) are plotted to visualize the contributions of the ORFs on the third and fourth LVs. ORFs with significant loadings (on LV3 and LV4, respectively) are indicated by red (positive loadings) and blue (negative loadings) circles, and the systematic ORF name provided.

In order to assess the contribution of biomass production, glucose consumption and ethanol production rates to the direction of the LVs, the loadings of response variables on the first four LVs are plotted in Figs 5A and 5B. Changes in glucose consumption and ethanol production rates in response to medium and gene deletion factors were modelled by the first two LVs and their trends were found to be similar to each other (Fig. 5A). Their loadings on LV1 are positive, and the transcriptome samples from ammonium limitation also score positively on LV1 (Fig. 4A); thus the model predicts an increase in the response variables under ammonium limitation as compared to glucose limitation. The response levels under ammonium limitation (Figs. 1A and 1B) are indeed higher when compared to those of the glucose-limited samples from the same mutant at an identical dilution rate. The ethanol and glucose load negatively on LV2, and they are expected to be affected positively by *hap4*Δ/*hap4*Δ deletion, which is indeed the case (Figs. 1B and 5A). The biomass has a positive score on LV2 and it is expected to be affected positively in the samples with positive scores on LV2, *i.e*. samples from *ho*Δ/*ho*Δ, but this prediction does not hold for ammonium-limited cases. However, it was previously explained that metabolic samples from *ho*Δ/*ho*Δ and *hap4*Δ/*hap4*Δ deletion mutants cannot be discriminated if the growth condition is ammonium-limited, as the deletions have no significant effect on the response variables if growth is N-limited.

All response variables have positive loadings on LV3 and LV4 (Fig. 5B), and therefore would be expected to have higher values in samples with positive scores on LV3 and LV4. Indeed, all response variables increased at the higher dilution rate (Fig. 1B). However, this behaviour cannot be predicted by the model as some of the scores of metabolic samples from higher dilution rates are not positive on LV3 and LV4 (Fig. 4D).

### Analysis of ORFs with significant contribution

Loadings of the ORFs on the latent variables were investigated to unravel the relationship between the transcriptome and response variables (Figs 5C–F). The ORFs with positive loadings on an LV are up-regulated in samples with positive scores on that LV while they are down-regulated in samples with negative scores. On the other hand, the ORFs with negative loadings on an LV are down-regulated in samples with positive scores on that LV.

The variance in LV1 represents the differences due to the medium factor. The genes with positive loadings on LV1, which are up-regulated under ammonium limitation when compared to glucose limitation, are expected to be the genes that mediate the increase in biomass production, glucose consumption, and ethanol production rates. Similarly, the genes with negative loadings on LV1 are most likely to be the genes that are up-regulated under glucose limitation causing the decrease in these response variables. The ORFs with positive loadings on LV2 are up-regulated in *ho*Δ/*ho*Δ deletion mutants, since samples from such mutants have positive scores on LV2. These ORFs are expected to mediate the changes in the rates of biomass production, glucose consumption and ethanol production in the *hap4*Δ/*hap4*Δ deletion mutants as compared to *ho*Δ/*ho*Δ deletion mutants.

The ORFs with significant positive or negative loadings (confidence interval 99.999%; Student's t < 1 × 10^-5^) on LVs are listed in Table 4. In addition, the ORFs listed under each LV group were analysed using SGD Gene Ontology Mapper [20], and the corresponding five cellular processes with lowest p-values among each group of ORFs are listed in Table 4.

The ORFs with significant positive loadings on the first latent variable (LV1+) are involved in hexose transport, these ORFs are up-regulated under ammonium limitation in comparison to carbon limitation. Up-regulation of the hexose transport pathway may be the first step of the mechanism to increase biomass production, ethanol production and glucose consumption rates under nitrogen limitation. The ORFs that are down-regulated under ammonium limitation as compared to glucose limitation are the genes that are active in oxidative phosphorylation, generation of precursor metabolites and energy (LV1-). The high glucose concentration in the ammonium-limited culture should repress the expression of genes acting on the respiratory pathways (oxidative phosphorylation). Repression of respiration, in turn, would cause the fermentation pathway to be activated and ethanol production to be enhanced.

The genes that were down-regulated due to the *hap4*Δ/*hap4*Δ deletion when compared to standart strain *ho*Δ/*ho*Δ mainly have roles in respiration and phosphate metabolism (LV2+). Consequently, the *hap4*Δ/*hap4*Δ deletion causes respiratory deficiency under the conditions studied, and fermentation is the only route for glucose metabolism. The higher glucose consumption and ethanol production rates achieved provide further confirmation that Hap4p plays a major role in the switch mechanism from respiration to fermentation. The genes that were up-regulated in response to the *hap4*Δ/*hap4*Δ deletion (LV2-) were involved in regulation of carbohydrate biosynthesis, indicating the propensity of the cells to convert excess carbon into storage molecules if the carbon source cannot be respired. While this theory explains the results from glucose-limited case perfectly, the effect of *hap4*Δ/*hap4*Δ deletion is not apparent in ammonia-limited conditions as the high glucose levels in the N-limited medium repress respiration, quite independently from the respiratory deficiency caused by the *hap4*Δ/*hap4*Δ deletion. Thus the metabolic variables behave similarly in *ho*Δ/*ho*Δ and *hap4*Δ/*hap4*Δ mutants growing under ammonium limitation, and the insignificant variation in these variables cannot be estimated by the model, as discussed previously.

The ORFs up-regulated at the higher dilution rate (LV3+) are the genes that act in ribonucleotide metabolism. Up-regulation of these ORFs mediates the increase in biomass production rate, and the consequent increases in ethanol production and glucose consumption rates. The GO terms common among the ORFs down-regulated at the lower dilution rate (LV3-) are related to reproduction mechanisms. The significance of these terms is quite low (p ~10^-2^); however, up-regulation of these mechanisms at the lower growth rate is an interesting phenomenon that remains to be explained.

The number of genes given in groups LV1- and LV2+ (Table 4) that are members of the GO terms "generation of precursor metabolites and energy" and "oxidative phosphorylation" have high significance (p < 1.0 E^-12^). The unknown ORFs that appear in the same group as these genes may also be members of the functional categories denoted by the over-represented GO terms.

## Conclusion

The use of formal experimental design allowed the analyses that we performed to discriminate between the effects that the growth medium, dilution rate, and the deletion of specific genes had on the transcriptome and metabolite profiles. The PLS method was applied to metabolic and transcriptomic data to gain insight into the changes in metabolism due to three factors (growth medium, dilution rate and gene deletion). The method enabled extraction of the following information from these data sets:

1. Discrimination of the effects of the above factors on transcriptome and metabolic data.

2. Modelling of metabolic data as a function of transcriptome data and elucidation of the extent of congruence between these two data sets.

3. Identification of ORFs that mediate the changes in metabolic data in response to perturbations.

In cases where the number of variables in the metabolic data is much higher, the PLS method will help in the identification of metabolites that are affected by the conditions applied and the genes that mediate the effects of the conditions. The unknown genes can be annotated using this methodology and studies towards product maximization can be conducted by identifying the genes and pathways that are responsible for the changes in formation of metabolic products.

## Methods

### Experimental materials and methods

Deletion strains of *S. cerevisiae *with genomic background *BY4743 *(*MATa*/*MAT*α *his3*Δ1/*his3*Δ1 *leu2*Δ0/*leu2*Δ0 *lys2*Δ0/*LYS2 MET15*/*met15*Δ0 *ura3*Δ0/*ura3*Δ0) from the Yeast Genome Deletion Project library [21] were used. The *ho*Δ/*ho*Δ deletant is commonly used as a standard strain in control experiments since the deletion has no measurable impact on either flux (growth rate, [22]) or the metabolome [23]. The absence of the *HO *or *HAP4 *genes in a strain's genome was verified using PCR-based methods.

Mineral media, supplemented with trace elements and vitamins were used [22]. The compositions of the media were as follows: KH_2_PO_4 _(2 g/l), MgSO_4_·7H_2_0 (0.55 g/l), NaCl (0.1 g/l), CaCl_2_·2H_2_O (0.09 g/l), Uracil (0.02 g/l), L-Histidine (0.02 g/l), L-Leucine (0.1 g/l), ZnSO_4_·7H_2_O (0.7 × 10^-4 ^g/l), CuSO_4_·5H_2_O (0.1 × 10^-4 ^g/l), H_3_BO_3 _(0.1 × 10^-4 ^g/l), KI (0.1 × 10^-4 ^g/l), FeCl_3_·6H_2_O (0.5 × 10^-4 ^g/l), inositol (0.12 g/l), thiamine/HCl (0.014 g/l), pyridoxine (0.004 g/l), Ca-pantothenate (0.004 g/l), biotin (0.0003 g/l).

For glucose-limited medium, 3.13 g/l (NH_4_)_2_SO_4 _and 2.5 g/l glucose were added to the medium described above. For ammonium-limited medium 0.46 g/l (NH_4_)_2_SO_4 _and 20 g/l glucose were added to medium described above.

The fermentors were autoclaved, and the media were filter-sterilized prior to inoculation. Pre-cultures (10 ml) were grown overnight in G418-containing YPD medium and used to inoculate the fermentors. The medium was fed at a constant flow rate. Temperature and pH of chemostats with 1L working volume were kept constant at 30°C and 4.5 respectively, and the oxygen content was maintained at saturation.

Homozygous *ho*Δ/*ho*Δ *and hap4*Δ/*hap4*Δ deletion strains were grown both in glucose-limited and ammonium-limited media in separate experiments. The experiments were started at a dilution rate of 0.1 h^-1 ^and, after samples had been collected, the dilution rate was shifted to 0.2 h^-1^. The samples were collected at steady state after three residence times, and total RNA extraction was carried out. Yeast Genome S98 arrays were used for hybridizations as described by the manufacturer (Affymetrix, USA, 2003, [24]). Supernatants were analyzed enzymatically for glucose and ethanol content (using kits from Boehringer-Mannheim, Germany).

Samples (5 ml) were centrifuged in pre-weighed tubes, dried at 80°C overnight and re-weighed to determine the dry weight of biomass.

### Experimental design

Factorial experimental design is used to reveal the effects of various factors on the output of a system. For an experiment set with "a" levels of "k" factors, a^k ^experiments are needed to cover all possible combinations. The 2^3 ^factorial design used in this work is given in Table 1. Eight experiments were conducted to investigate all combinations of the factors. Levels of the factors and the corresponding experimental conditions are given in Table 2.

The abbreviations used for the homozygous *ho*Δ/*ho*Δ and *hap4*Δ/*hap4*Δ mutants deletion strains are "*ho*Δ" and "*hap4*Δ", respectively. The "G" and "N" are the abbreviations for the glucose and ammonium-limited cases, while "1" and "2" are used for dilution rates 0.1 h^-1 ^and 0.2 h^-1^, respectively. In the Figures, "Δ" is omitted and the abbreviations *ho *and *hap4 *represent the deletion mutants.

### Linear modelling

The linear model for a factorial experiment with three factors is as follows [25]:

*y*_*ijk *_= μ + τ_*i *_+ β_*j *_+ γ_*k *_+ ε_*ijk *_    (1)

where i, j, k: indices of the levels of the factors; i = 1, 2, ..., a; j = 1, 2, ..., b; k = 1, 2, ..., c; μ: mean of the outputs; y: simulated value of the output variable; τ, β, γ: effects of the factors; ε: random error. In the present experimental design, each factor has two levels; thus, a = b = c = 2. The output variable y represents the expression level of an ORF on a log _2 _basis.

The linear model in Eq.(1) was used to estimate the coefficients to describe the expression level of each ORF. The coefficients obtained in each case are the "effects" of the factors on the expression of the ORF modelled. A positive effect made by a factor indicates that the ORF is up-regulated at Level (+) when compared to Level (-) of that factor. Similarly, a negative effect indicates down-regulation of the ORF at Level (+) when compared to Level (-).

P-values of the factors were calculated using the ratio of variation sum of squares to error sum of squares in order to indicate the significance of the correlation between the gene expression and the factor.

### Partial least squares

In industrial processes, large sets of process data are collected by computerized control and monitoring systems. Multivariate data analysis methods have emerged to compensate for the need for data reduction towards understanding the nature of the process and fault diagnosis. Partial least squares (projection to latent structures – PLS) is a statistical method that was proposed for process analysis, monitoring and diagnosis [26-28]. Later on, this method was employed as one of the standard tools of chemometrics in analytical chemistry [29].

In PLS methodology, the independent "cause" matrix X and the dependent "response" matrix Y are regressed and modelled simultaneously. The columns of these matrices represent the variables (genes and response variables in X and Y, respectively) and rows represent the samples. The linear model is:

Y = XB + E     (2)

where B is the regression vector and E is the residual matrix.

Projection of the original data set X to a new space with reduced dimensions is made by the loading matrix (p) and the observations are represented by the score matrix (t) in the new space. Decomposition of the data matrix X into the score matrix (t), the loading matrix (p) and the residual matrix (e) is as follows:

X = tp^*t *^+ e     (3)

where the superscript "*t*" denotes the transpose of the matrix p. Columns of p and t matrices correspond to the latent variables (LVs), which lie in the direction of the maximum variation that remains in the data after removal of the variation explained by the previous LV. The residual matrix (e) represents the variation that remains unrepresented in the t and p matrices. Similarly, the response matrix Y is decomposed as:

Y = uq^*t *^+ f     (4)

The score vectors (vectors of u) and the loading vectors (vectors of q) correspond to LVs. The residuals are given by the f matrix. A linear inner relation also exists between the matrices t and u, where ū denotes the matrix of estimated values of u:

ū = bt     (5)

The optimal number of LVs to be included in the model depends on the amount of variation explained by the LVs which are in descending order of the variation they explain. One criterion for the selection of an optimal number of LVs is to set a threshold value for the variation. Then, a sufficient number of LVs is included in the model to represent the threshold variation, and the rest of the variation remains in the residual matrix. Cross-validation is another criterion where the analysis is performed with a subset of the data and the rest of the data set is used to determine the prediction power of the model. Then, the number of LVs that results in minimum prediction error sum of squares (PRESS) is selected.

## Authors' contributions

The biological problem was conceived by BK and SGO; the experiments were executed by PP and AH. The PLS approach was suggested by KOU who assisted PP in the analyses. The manuscript was written by PP, BK, KOU and ZIO, and was revised by SGO. All authors read and approved the final version.

## Figures and Tables

**Table 1:** 2^3 ^Factorial Experiment Design

Run	Deletion	Medium	Rate	Sample Name
1	-	-	-	*ho*Δ G1^a^
2	+	-	-	*hap4*Δ G1
3	-	+	-	*ho*Δ N1
4	+	+	-	*hap4*Δ N1
5	-	-	+	*ho*Δ G2
6	+	-	+	*hap4*Δ G2
7	-	+	+	*ho*Δ N2
8	+	+	+	*hap4*Δ N2

^a ^In the Figures, the symbol 'Δ' is omitted from the sample names.

**Table 2:** Factors and Experimental Conditions

Factor	Level (-)	Level (+)
Deletion	Homozygous diploid, *ho*Δ/*ho*Δ	Homozygous diploid, *hap4*Δ/*hap4*Δ
Medium	Glucose-limited	Ammonium-limited
Dilution Rate	0.1 h^-1^	0.2 h^-1^

**Table 3:** Proportion of the variation explained by each latent variable

LV	% Variation (X)	Cumulative % variation (X)	% Variation (Y)	Cumulative % variation (Y)
1	56.0	56.0	73.7	73.7
2	13.6	69.6	13.1	86.8
3	25.0	94.6	2.8	89.7
4	1.7	96.3	8.2	97.9
5	1.4	97.6	2.1	99.9
6	1.4	99.0	0.0	100.0
7	1.0	100.0	0.0	100.0

**Table 4:** ORFs and GO terms with highest contributions to the LVs

LV	ORFs with significant loadings	Biological Process GO Terms	P-value
LV1+	HXT1, MNT4, HXT3, YER028C, YJL132W, YGL157W, ALD1, ZRT2	hexose transport	1.8E-04
		monosaccharide transport	1.8E-04
		carbohydrate transport	5.2E-04
		transport	7.4E-02
		establishment of localization	7.7E-02
LV1-	GSY1, MBR1, ISF1, GDB1, MAL33, QCR8, GLG1, PIG1, YDL157C, CBP4, GPH1, HXK1, GAC1, YPR196W, YLR327C, PRX1, QCR9, PCL7, MAL31, BAP2, INH1, MRK1, YOL053W, YKL187C, YMR103C, MTH1, MCR1, YGR243W, PRS2, ROM1, COX8, COX4, YJR008W, YNL274C, HOR2, COX7, YPL099C, ATP18, QCR10, CNM67, ATP5, ACN9, COX12, COX6	generation of precursor metabolites and energy	1.1E-21
		energy derivation by oxidation of organic compounds	4.4E-17
		oxidative phosphorylation	2.0E-13
		electron transport	1.7E-11
		ATP synthesis coupled electron transport (sensu Eukaryota)	1.9E-10
LV2+	QCR8, PRX1, QCR9, INH1, MCR1, COX8, COX4, COX12, COX6, COX7, ATP18, QCR10, ATP5, AMS1, HAP4, RPM2, PHM8, FBP26, ATP15, YMR034C, YOR220W, TUF1, COR1, ATP3, YNL122C, ATP7, ATP17, ATP20, HXT1, YER028C, YJL132W	oxidative phosphorylation	2.7E-25
		generation of precursor metabolites and energy	5.7E-18
		phosphorylation	6.0E-18
		phosphorus metabolism	4.1E-16
		phosphate metabolism	4.1E-16
LV2-	PIG1, BAP2, MRK1, PRS2, UBP14, MKC7	regulation of carbohydrate biosynthesis	8.1E-05
		regulation of carbohydrate metabolism	2.1E-04
		regulation of cellular biosynthesis	7.4E-04
		regulation of biosynthesis	7.4E-04
		carbohydrate biosynthesis	1.2E-03
LV3+	PRS2, CBP4, RPL7A, YOR314W, ALD1, ATP5, COX6	purine ribonucleotide biosynthesis	5.6E-04
		purine ribonucleotide metabolism	6.2E-04
		ribonucleotide biosynthesis	6.2E-04
		ribonucleotide metabolism	6.8E-04
		purine nucleotide biosynthesis	7.1E-04
LV3-	PIG1, ROM1, CNM67, YBL112C, MSC2, YOL153C, UBI4, RAD2, CHS1, MNT4, ZRT2, PRX1, AMS1, PHM8, FBP26, YMR034C, YOR220W, HXT1, YJL132W	asexual reproduction	1.9E-02
		cell budding	1.9E-02
		carbohydrate metabolism	1.9E-02
		reproduction	2.9E-02
		cell homeostasis	3.4E-02
LV4+	MSC2, PRX1, AMS1, PHM8, YMR034C, HXT1, MTH1, YPL099C, TRS23, CYC7, ZRG17, YLR431C, GPG1, YFL034W, PKH1, HXT3, YER028C	monosaccharide transport	1.2E-05
		hexose transport	1.2E-05
		carbohydrate transport	5.5E-05
		transport	6.5E-04
		establishment of localization	7.4E-04
LV4-	CNM67, MRK1, MKC7, YDL157C, YDR119W, QCR8, QCR9, INH1, COX8, COX4, HAP4, ALD1	oxidative phosphorylation	7.6E-09
		phosphorylation	5.0E-08
		ATP synthesis coupled electron transport (sensu Eukaryota)	6.7E-08
		ATP synthesis coupled electron transport	6.7E-08
		electron transport	1.6E-07

Student's t-test was applied to loadings of the ORFs and only those ORFs with t < 1 × 10^-5 ^(within confidence interval 99.999%) are listed in the Table. GO Mapping was applied to these ORFs and only the top five significant biological process terms are given. (+) and (-) signs indicate positive and negative loadings of the ORFs, respectively.
